# Causes of Revision of Total Knee Arthroplasties in a Tertiary Hospital in Brazil

**DOI:** 10.1055/s-0042-1757304

**Published:** 2024-12-07

**Authors:** Alan de Paula Mozella, Hugo Alexandre de Araújo Barros Cobra, Sandra Tie Nishibe Minamoto, Rodrigo Salim, Ana Carolina Leal

**Affiliations:** 1 Departamento de Ortopedia e Traumatologia, Centro de Cirurgia do Joelho, Instituto Nacional de Traumatologia e Ortopedia (INTO), Rio de Janeiro, RJ, Brasil.; 2 Departamento de Ortopedia e Anestesiologia, Faculdade de Medicina de Ribeirão Preto, Universidade de São Paulo (USP), São Paulo, SP, Brasil.; 3 Divisão de Ensino e Pesquisa, Instituto Nacional de Traumatologia e Ortopedia (INTO), Rio de Janeiro, RJ, Brasil.

**Keywords:** arthroplasty, arthroplasty, replacement, knee, infections, knee joint

## Abstract

**Objective** To identify the causes of revision of total knee arthroplasty in a referral center in Brazil.

**Methods** This is a case series, with 80 patients undergoing revision surgery for total knee arthroplasty (RTKA) at a referral center for knee surgery, between August 2019 and November 2021, with a mean age of 69.6 years. Of these patients, 60.23% were female and 39.77% were male. The average body mass index (BMI) was 30.23 kg/m ^2^ . The causes of TKA failure were defined as: periprosthetic infection according to the 2018 International Consensus Meeting criteria, ligament instability, range of motion limitation, periprosthetic fracture, malalignment, aseptic loosening, pain due to non-replacement of the patellar cartilage, polyethylene wear, fracture of implants, insufficiency of the extensor mechanism.

**Results** Periprosthetic joint infection (PJI) was the main cause of revision total knee arthroplasty (TKA), corresponding to 47.73% of cases. Aseptic loosening of one or more components represented the second most frequent reason for TKA failure, accounting for 35.23% of revisions. Range of motion limitation represented the third most frequent cause, accounting for 5.68% of surgeries. Instability was the fourth most frequent reason for RTKA, occurring in 4.55% of patients. The other causes of revision were: periprosthetic fracture (3.41%), failure due to rupture of the extensor mechanism (2.27%), and pain attributed to non-replacement of the patellar cartilage (1.14%).

**Conclusions** Periprosthetic joint infection was the most frequent cause of TKA revision in our series. Other reasons for TKA failures were, in descending order: aseptic loosening, limited range of motion, and instability.

## Introduction

Epidemiological data show that between 6 and 12% of total knee arthroplasties (TKA) evolve to failure, requiring revision surgery in the first 10 years. 1 Other recent studies have shown an increase in the number of revision surgeries of TKA (RTKA) performed in recent years. 2 3 4 5 There was a 144% increase in the number of RTKA performed in Germany between 2004 and 2014. 6 It is estimated that, for the year 2050, in the same country, there will be an increase of about 90% in the number of revision surgeries of knee arthroplasties. 4 In the United States, the increase in the number of such procedures was of 39% between 2006 and 2010. 7 And future estimates point to a continuous increase in the number of these surgeries by the year 2030. 8

The etiology for increasing the number of RTKAs is, in fact, multifactorial, being influenced both by factors related to technical issues and evolution of implants and instruments, as well as by factors related to patients. 9 10 An example of this, the wear of the polyethylene component was a frequent cause of revision in the past; however, changes in the sterilization process of these components culminated in a significant reduction in the number of revisions for this reason. 11 12 13 At the same time, the expansion of primary prosthesis indications, especially for younger patients, and the higher incidence of obesity in the world population may, at least partially, also justify potential changes in the failure patterns of modern arthroplasties. 9 14 15 Meehan et al. 14 demonstrated that the risk of septic failure was 1.81 times higher in patients under 50 years of age when compared to patients older than 65 years. Similarly, among younger patients, the risk of revision for aseptic failure was 4.7 times higher. 14

Knee prosthesis revision surgeries are procedures of high complexity, performed in a limited number of hospitals and, consequently, that require greater expenditure of technical and economic resources. 16 17 Compliance with technical principles is essential to obtain satisfactory results in this type of surgery. 18 Thus, preoperative planning, failure mode identification and adequate treatment of bone defects are of fundamental importance for obtaining satisfactory and long-lasting clinical results in this type of surgery. 18 19

In this context, the identification of the cause of implant failure is significantly important for planning and obtaining satisfactory results. 20 Several series have demonstrated changes in the causes of TKA failure over the years, possibly related to evolutions in implant design, as well as instrumental improvements. 11 12 Thus, this study aims to identify the causes of RTKA in a reference center in Brazil.

## Materials and Methods

This is a series of cases of patients undergoing RTKA at the Center for Specialized Attention in Knee Surgery of Instituto Nacional de Traumatologia e Ortopedia (INTO) Jamil Haddad, in the period between August and November 2019 (CAAE- 20309419.0.0000.5273.). There was no age limit, no restriction on the gender of the participants.

During the study period, 88 arthroplasty revision surgeries were performed. One patient underwent revision of the unicompartmental implant of the knee and was therefore excluded. Seven surgeries were excluded from the analysis because it was the second revision surgery. Thus, after the exclusion criteria, 80 surgeries (80 patients) were analyzed. The mean age of the patients in the procedures was 69.6 years (standard deviation (SD) = 9.85 years; variation = 29–87 years). Of these patients, 60.23% were female and 39.77% were male. The mean body mass index (BMI) was 30.23 kg/m ^2^ (SD = 5.92; variation = 18.20–42.46 kg/m ^2^ ).

Arthroplasty review was defined as reoperation after performing primary TKA in which it was necessary to add or replace one or more prosthetic components. Thus, patients submitted to reoperations, however without the addition or replacement of one or more primary prosthetic components, were excluded from the analysis. Patients submitted to unicompartmental knee implant (UKI) revision surgery were also excluded from the analysis, as well as patients submitted to the second revision surgery.

The following data were collected from medical records: age, gender, and BMI. Laboratory tests with analysis of hemo-sedimentation velocity (HSV), C-reactive protein (CRP), and D-dimer were performed in all patients on the day before surgery, as well as range of motion measurement using a goniometer by the orthopedist in training in the knee surgery improvement course at the institution. Radiographs of the knee at anteroposterior incidences with bipodal, profile, and axial support in 30 degrees were performed on the day before surgery. During surgery, synovial fluid was collected for the following analyses: total leukocyte count, polymorphonuclear percentage, leukocyte esterase, and culture in a blood culture vial. Six samples of bone tissue were also collected for extended tissue culture for 14 days and 2 periprosthetic membrane samples for histopathological examination.

In the surgical report, the following information were analyzed: surgical access performed, implant system used, Anderson Orthopaedic Research Institute (AORI) classification of bone loss, use of trabeculated metal cones and/or tissue bank grafts for the treatment of defects.

Regarding the surgical access used, the surgery was performed by conventional medial parapatellar access in 70 surgeries (87.5%). Extended access was performed in 10 surgeries (12.5%), with tibia anterior tuberosity (TAT) osteotomy in 7 cases (8.75%), quadriceps turndown in two cases (2.5%), and clipping of the thigh reskin (snip) in one patient (1.25%).

Implants with two different systems—Legacy Constrained Condylar Knee (Zimmer Biomet, Warsaw, IN, USA) or Legion Revision Knee (Smith & Nephew plc, London, UK)—were used in 40 surgeries (50%). In 24 surgeries (30%), constrictor implants (Rotating Hinge Knee – Zimmer Biomet) were used. Spacers were implanted in 14 patients (17.5%). In 2 patients (2.5%), only patellar implants were used.

Medical documentation of bone defects was incomplete in five surgeries. Thus, when analyzing 75 patients, the presence of bone defects was identified in 70% of the patients. The distribution and classification of bone failures are shown in Table 1 . Tantalum metaphysary cones were necessary for the treatment of bone defects in 21 patients (26.25%). Of these, tissue bank grafting was also used in nine surgeries. In all, the homologous graft was used in 13 patients (16.25%).

**Table 1:** Distribution of bone defects

Classification of defects	Tibia n (%)	Femur n (%)
Flawless	15 (20%)	16 (21.3%)
1	20 (26.7%)	15 (20%)
2a	19 (25.3%)	15 (20%)
2b	9 (12%)	8 (10.7%)
3	12 (16%)	21 (28%)

Source: Research data (INTO).

The cause of RTKA was defined by the surgeon responsible for the procedure based on the following criteria: all patients were submitted to evaluation according to the criteria of the 2018 International Consensus Meeting (ICM 2018). 21 22 The diagnosis of periprosthetic infection was made when at least one of the major criteria was present, that is, the presence of a fistula communicating with the joint or presence of the same pathogen in two or more cultures of periimplant tissues. The diagnosis of periprosthetic infection using the lower criteria was confirmed when the score of the sum of the criteria was greater than or equal to six when the parameters of the tests recommended by the ICM 2018 were observed. In cases defined with aseptic failures, the surgeon in charge, after analysis of the physical examination, imaging and laboratory examinations defined the reason for failure between the following possible: ligament instability (when presence of dislocation or subluxation of prosthetic components, or when pathological ligament opening greater than 5 mm during physical examination, or ligament laxity greater than 5 mm in varus or varus stress tests during physical examination or ap radiographs of the knee with load), limitation of range of motion (when less than 50 degrees associated with physical disability declared by the patient), periprosthetic fracture, poor alignment, aseptic loosening, pain due to non-replacement of patellar cartilage, polyethylene wear, fracture of implants, or insufficiency of the extensor mechanism.

## Results

**Fig. 1:**
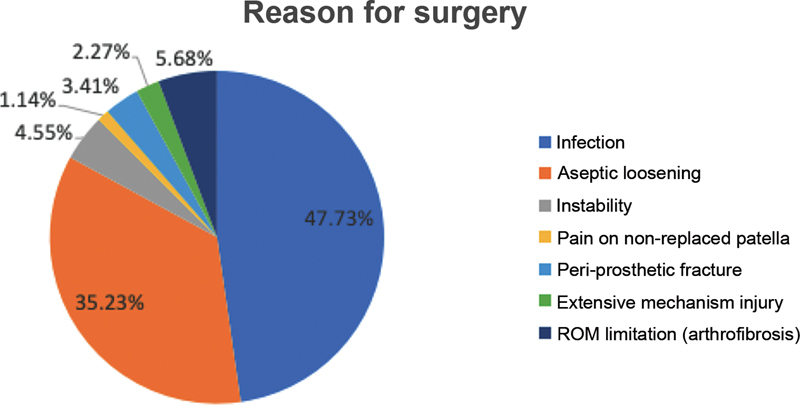
Distribution of the causes of failure of total knee arthroplasty. Causa da Cirurgia = Reason for surgery, Infecção = Infection, Soltura Asséptica = Aseptic loosening, Instabilidade = Instability, Dor em patela não substituída = Pain on non-replaced patella, Fratura periprotética = Peri-prosthetic fracture, Lesão do mecanismo extensor = Extensive mechanism injury, Limitação da ADM (artrofibrose) = ROM limitation (arthrofibrosis)

Periprosthetic joint infection (PJI) was the main cause of RTKA, corresponding to 47.73% of cases. The aseptic loosening of one or more components represented the second most frequent cause of failure, resulting in 35.23% of revisions. Limitation of the range of motion represented the 3 ^rd^ most frequent cause, accounting for 5.68% of surgeries. Instability was the 4 ^th^ most frequent cause of failure, occurring in 4.55% of patients. The other causes of revision were: periprosthetic fracture (3.41%), insufficiency due to rupture of the extensor mechanism (2.27%), pain attributed to non-replacement of patellar cartilage (1.14%) ( Fig. 1 ).

## Discussion

The main finding of our study was to identify that the main cause of failure in our series of primary TKAs was periprosthetic infection. Other reasons that led to the need for revision were, in decreasing order, aseptic loosening, limitation of the range of motion, and joint instability. Similarly, several recent studies have demonstrated periprosthetic infection as the most frequent mechanism of failure of primary prostheses, reaching 20 to 37.7% of surgeries. 2 23 24 25 In the study by Evangelopoulos et al., 24 periprosthetic infection was the main mechanism of failure with an incidence of 26.3%. However, when analyzing only patients submitted to the second revision surgery, the authors identified that the incidence of periprosthetic infection remained the most frequent reason for TKA failure with approximately 50% of cases. We believe that this high incidence of revisions due to septic failure observed in our study may have been influenced, at least partially, by the fact that the surgeries were performed in the COVID-19 pandemic period. In this scenario, in several months, only emergency surgeries were performed, such as acute periprosthetic infections and, consequently, subsequent increase in the demand for reimplantation after treatment of infection in two times.

Aseptic loosening represented the 2 ^nd^ most frequent reason for implant failure in our series, occurring in approximately 35% of cases. Similarly, Bozic et al. 7 demonstrated that periprosthetic infection and aseptic loosening were the two most frequent reasons for TKA revision. When analyzing more than 60,000 primary surgeries, the authors identified septic failure in 25.1% of patients and aseptic loosening in 16.7% of cases. 7 Our results are in agreement with those of Koh et al. 26 and Evangelopoulos et al., 24 who demonstrated aseptic loosening as the 2 ^nd^ main cause of review, affecting, respectively, 32.7% and 25% of the failures. Siqueira et al., 1 when analyzing the arthroplasty registry of five countries, identified aseptic loosening as the first cause of revision, affecting 30% of surgeries. Several other studies have also reported aseptic loosening as the main mechanism of TKA failure with incidence ranging from 24 to 44%. 10 27 28 We identified that the incidence of aseptic loosening in our series is in accordance with the literature, although it is not the main mode of failure of our implants.

The limitation of the range of motion represented, in our series, the third most common indication for RTKA, with approximately 6% of surgeries. Similarly, in the study by Le et al., arthrofibrosis was the third most frequent indication for review, although the incidence presented corresponds to approximately three times that observed in our study. Pietrzak et al. 11 studied only the aseptic causes of revision and identified joint stiffness as the 2 ^nd^ leading cause of revision with an incidence of 27.5%. Meanwhile, Le 29 and Koh et al. 26 showed an incidence that only 2.5% of patients required TKA revision due to functional limitation imposed by movement arc restriction. Such differences can be explained, at least partially, by the various definitions of joint stiffness, as well as being influenced by the functional demand and expectations of patients. 30 31 32 We should also observe that, in our series, we included only patients submitted to revision of prosthetic components; thus, patients submitted to manipulation under narcosis after TKA or even reoperation with joint release were not analyzed, which could have modified the incidence if we analyzed reoperations after knee prosthesis. In a study conducted in Brazil evaluating short-term complications (up to 1 year), the authors identified that joint stiffness was the most frequent complication affecting 7.5% of patients undergoing primary knee arthroplasty. 33

In our study, component instability represented the 4 ^th^ cause of RTKA, with approximately 5% of surgeries. Other studies have demonstrated an incidence of instability ranging from 18.7 to 30%, constituting the 2 ^nd^ or 3 ^rd^ most frequent reason for TKA review. 10 28 29 However, in the works of Kasahara et al. 27 and Koh et al., 26 instability was identified in 9.3% and 6.5%, respectively. Post-TKA instability is usually indicated as a cause of early failure, that is, in implants with less than 2 years of evolution. 6 11 Thus, the reduction in the number of elective primary surgeries performed during the study period, due to the restrictions imposed by the COVID-19 pandemic, may justify, at least partially, the limited number of revisions due to post-TKA instability. Another potential explanation can be attributed to the waiting time for this type of surgery in the Brazilian public health service. Thus, any initial cases of instability can potentially evolve with loosening of the implants, thus hindering the primary diagnosis of component instability.

Despite the importance of our series reporting the distribution of the different causes of RTKA in the national scenario, our study has significant limitations. We believe that restrictions on elective surgeries imposed during part of the COVID-19 pandemic period may influence the incidence of some modes of failure, such as suspension of elective aseptic revisions in certain periods. Another important limitation refers to the retrospective nature of our analysis. Thus, several patients submitted to revision surgery had been operated on primarily in other hospitals. Thus, we had no control over the evolution time from the implantation of the components to the occurrence of failure requiring revision surgery, nor did we perform analyses of the different systems of primary prostheses implanted. Schroer et al. 10 indicate that 35.3% of knee arthroplasties fail in the first 2 years after implantation, being more often related to factors influenced by the surgeon than by the performance of implants. However, we understand that such information is of high relevance for the planning of the Brazilian health system in view of the increase in the number of cases of RTKA, given, the impacts regarding the need for hospital beds, financial resources, and technical training of the teams. Similarly, we believe that the high number of revision surgeries performed due to septic failure observed in our series alerts us to the need to expand the measures to prevent these serious complications.

Therefore, we believe that the adoption of preventive measures of postoperative infection, such as: optimization of preoperative clinical conditions, adoption of risk stratification scores and investigation of patients colonized by *Methicillin-resistant Sthapylococcus aureus* may impact on the reduction of the number of RTKA surgeries performed in our hospital.

## Conclusions

We identified periprosthetic joint infection as the most frequent cause of RTKA. The other TKA failure mechanisms were, in decreasing order: aseptic loosening, limitation of the range of motion, and instability. Periprosthetic fracture and extensor mechanism insufficiency were less frequent causes of primary prosthesis revision in our series.
