# Supplementary material for: Causes of Revision of Total Knee Arthroplasties in a Tertiary Hospital in Brazil
Source: Rev Bras Ortop (Sao Paulo). 2024 Dec 7;59(5):e696–701. doi: 10.1055/s-0042-1757304 (PMC11624922; doi:10.1055/s-0042-1757304)
Supplement: Related texts

# Research article: Causas de revisão de artroplastias totais do joelho em um hospital terciário no Brasil

**Keywords:** articulação do joelho, artroplastia, artroplastia do joelho, infecções

## Abstract

**Objetivo** Identificar as causas de revisão de artroplastia total do joelho em um centro de referência no Brasil.

**Métodos** Trata-se de uma série de casos, com 80 pacientes submetidos à cirurgia de revisão de artroplastia total do joelho (RATJ) em um centro de referência em cirurgia do joelho, no período entre agosto de 2019 e novembro de 2021, com média de idade de 69,6 anos. Desses pacientes, 60,23% eram do sexo feminino e 39,77% do sexo masculino. O índice de massa corporal (IMC) médio foi de 30,23 kg/m ^2^ . As causas de falha da ATJ foram definidas como: infecção periprotética conforme critérios do *2018 Internacional Consensus Meeting* , instabilidade ligamentar, limitação de arco de movimento, fratura periprotética, mal alinhamento, soltura asséptica, dor devido à não substituição da cartilagem patelar, desgaste do polietileno, fratura dos implantes, insuficiência do mecanismo extensor.

**Resultados** A infecção articular periprotética (IAP) foi a principal causa de revisão de artroplastia total do joelho (ATJ), correspondendo a 47,73% dos casos. A soltura asséptica de um ou mais componentes representou o segundo motivo mais frequente para causa de falha de ATJ, acarretando 35,23% das revisões. Limitação do arco de movimento representou a terceira mais frequente causa, sendo responsável por 5,68% das cirurgias. Instabilidade foi a 4ª razão mais frequente para RATJ, ocorrendo em 4,55% dos pacientes. As demais causas de revisão foram: fratura periprotética (3,41%), insuficiência por ruptura do mecanismo extensor (2,27%), dor atribuída à não substituição da cartilagem patelar (1,14%).

**Conclusões** A infecção articular periprotética foi a causa mais frequente de revisão de ATJ em nossa série. Outras razões de falhas de ATJ foram, em ordem decrescente: soltura asséptica, limitação do arco de movimento e instabilidade.

## Introdução

Dados epidemiológicos demonstram que entre 6 e 12% das artroplastias totais do joelho (ATJ) evoluem para falha, necessitando de cirurgia de revisão nos primeiros 10 anos. 1 Outros estudos recentes evidenciaram aumento do número de cirurgias de revisão da artroplastia total do joelho (RATJ) realizadas nos últimos anos. 2 3 4 5 Foi observada elevação de 144% do número de RATJs realizadas na Alemanha entre os anos de 2004 e 2014. 6 Estima-se, para o ano de 2050, no mesmo país, ocorra uma elevação de cerca de 90% do número de RATJs. 4 Nos Estados Unidos, o aumento do número desse tipo de procedimento foi de 39% entre 2006 e 2010. 7 E estimativas futuras apontam para contínua elevação do número dessas cirurgias até o ano de 2030. 8

A etiologia para o aumento do número de RATJs é, na realidade, multifatorial, sendo influenciada tanto por fatores relacionados às questões técnicas e de evolução dos implantes e instrumentais quanto por fatores relativos aos pacientes. 9 10 Exemplo disso, o desgaste do componente de polietileno era causa frequente de revisão no passado; entretanto, modificações do processo de esterilização desses componentes culminaram com significativa redução do número de revisões por essa razão. 11 12 13 Concomitantemente, a expansão das indicações da prótese primária, sobretudo para pacientes mais jovens e a maior incidência de obesidade na população mundial podem, pelo menos parcialmente, justificar, também, potenciais modificações dos padrões de falha das modernas artroplastias. 9 14 15 Meehan et al. 14 demonstraram que o risco de falha séptica foi 1,81 vezes maior em pacientes com idade inferior a 50 anos quando comparados com pacientes maiores de 65 anos. De modo similar, entre os pacientes mais jovens, o risco de revisão por falha asséptica foi 4,7 vezes maior. 14

As cirurgias de revisão de prótese do joelho são procedimentos de alta complexidade, realizados em limitado número de hospitais e, consequentemente, que demandam maior dispêndio de recursos técnicos e econômicos. 16 17 A observância dos princípios técnicos é fundamental para obtenção de resultados satisfatórios nesse tipo de cirurgia. 18 Assim, o planejamento pré operatório, a identificação do modo de falha e o adequado tratamento dos defeitos ósseos são de fundamental importância para obtenção de resultados clínicos satisfatórios e duradouros nesse tipo de cirurgia. 18 19

Neste contexto, a identificação da causa de falha dos implantes apresenta significativa importância para o planejamento e obtenção de resultados satisfatórios. 20 Diversas séries demonstraram modificações das causas de falha de ATJ ao longo dos anos, possivelmente, relacionado às evoluções no desenho dos implantes, assim como, melhorias no instrumental. 11 12 Desse modo, este estudo tem como objetivo identificar as causas de revisão de artroplastia total do joelho em um centro de referência no Brasil.

## Materiais e Métodos

Trata-se de uma série de casos de pacientes submetidos à cirurgia de RATJ no Centro de Atenção Especializada em Cirurgia do Joelho do Instituto Nacional de Traumatologia e Ortopedia (INTO) Jamil Haddad, no período compreendido entre agosto de 2019 e novembro de (CAAE- 20309419.0.0000.5273.). Não houve limite de idade, nem restrição quanto ao sexo dos participantes.

No período do estudo, foram realizadas 88 cirurgias de revisão de artroplastia. Um paciente foi submetido à revisão de implante unicompartimental do joelho, sendo, portanto, excluído. Sete cirurgias foram excluídas da análise por se tratar de segunda cirurgia de revisão. Desse modo, observados os critérios de exclusão, 80 cirurgias (80 pacientes) foram analisadas. A média de idade dos pacientes no procedimento foi de 69,6 anos (desvio-padrão [DP] = 9,85 anos; variação = 29–87 anos). Desses pacientes, 60,23% eram do sexo feminino e 39,77% do sexo masculino. O índice de massa corporal (IMC) médio foi de 30,23 kg/m ^2^ (DP = 5,92; variação = 18,20–42,46 kg/m ^2^ ).

Revisão de artroplastia foi definida como reoperação após a realização da ATJ em que foi necessário a adição ou substituição de um ou mais componentes protéticos. Assim, pacientes submetidos a reoperações, contudo, sem a adição ou substituição de um ou mais componentes protéticos primários foram excluídos da análise. Foram, ainda, excluídos da análise: pacientes submetidos à cirurgia de revisão de implante unicompartimental do joelho (AUJ), pacientes submetidos à segunda cirurgia de revisão.

Foram coletados do prontuário médico os seguintes dados: idade, sexo eIMC. Exame laboratorial com análise da velocidade de hemossedimentação (VHS), proteína C reativa (PCR) e D-dímero foram realizados em todos os pacientes no dia anterior à cirurgia, assim como, aferição pelo médico ortopedista em treinamento no curso de aperfeiçoamento em cirurgia do joelho na instituição, do arco de movimento com utilização de um goniômetro. Radiografias do joelho nas incidências anteroposterior com apoio bipodal, perfil e axial em 30 graus foram realizados no dia que antecedia a cirurgia. Durante o ato cirúrgico foi coletado líquido sinovial para as seguintes análises: contagem total de leucócitos, percentual de polimorfonuclear, esterase leucocitária e cultura em frasco de hemocultura. Foram coletadas, ainda, 6 amostras de tecido ósseo para realização de cultura de tecidos estendida por 14 dias e 2 amostras de membrana periprotética para exame histopatológico.

No relatório cirúrgico, as seguintes informações foram analisadas: acesso cirúrgico realizado, sistema de implantes utilizados, classificação Anderson Orthopaedic Research Institute (AORI) da perda óssea, utilização de cones de metal trabeculado e/ou enxertos de banco de tecido para o tratamento dos defeitos.

Quanto ao acesso cirúrgico utilizado, a cirurgia foi realizada por acesso parapatelar medial convencional em 70 cirurgias (87,5%). O acesso estendido foi realizado em 10 cirurgias (12,5%), sendo em 7 casos (8,75%) a osteotomia da tuberosidade anterior da tíbia (TAT), *turndown* quadríceps em 2 casos (2,5%) e recorte do reto da coxa ( *snip* ) em 1 paciente (1,25%).

Implantes semiconstritos de dois distintos sistemas – Legacy Constrained Condilar Knee (Zimmer Biomet, Warsaw, IN, EUA) ou Legion Revision Knee (Smith & Nephew plc, London, Reino Unido) – foram utilizados em 40 cirurgias (50%). Em 24 cirurgias (30%), foram utilizados implantes constritos rotatórios (Rotating Hinge Knee – Zimmer Biomet). Espaçadores foram implantados em 14 pacientes (17,5%). Em 2 pacientes foram utilizados apenas implantes patelares (2,5%).

A documentação médica dos defeitos ósseos foi incompleta em cinco cirurgias. Assim, ao analisarmos 75 pacientes, a presença de defeitos ósseos foi identificada em 70% dos pacientes. A distribuição e classificação das falhas ósseas são demonstradas na Tabela 1 . Cones metafisários de tântalo foram necessários para tratamento dos defeitos ósseos em 21 pacientes (26,25%). Desses, em 9 cirurgias também foi utilizado enxerto de banco de tecido. Ao todo, o enxerto homólogo foi utilizado em 13 pacientes (16,25%).

**Tabela 1:** Distribuição dos defeitos ósseos

Classificação dos defeitos	Tíbia n (%)	Fêmur n (%)
Sem defeito	15 (20%)	16 (21,3%)
1	20 (26,7%)	15 (20%)
2A	19 (25,3%)	15 (20%)
2B	9 (12%)	8 (10,7%)
3	12 (16%)	21 (28%)

Fonte: Dados da pesquisa (INTO).

A causa da RATJ foi definida pelo cirurgião responsável pelo procedimento baseado nos seguintes critérios: todos os pacientes foram submetidos à avaliação segundo os critérios do *2018* *International Consensus Meeting* (ICM 2018). 21 22 O diagnóstico de infecção periprotética foi realizado quando presente pelo menos um dos critérios maiores, isto é, presença de fístula comunicante com a articulação ou presença do mesmo patógeno em duas ou mais culturas de tecidos peri-implantes. O diagnóstico de infecção periprotética por meio dos critérios menores foi confirmada quando, observados os parâmetros dos testes preconizados pelo ICM 2018, a pontuação do somatório dos critérios foi maior ou igual a seis. Nos casos definidos com falhas assépticas, o cirurgião responsável, após análise do exame físico, exames de imagem e laboratorial definiu a razão da falha ente as seguintes possíveis: instabilidade ligamentar (quando presença de luxação ou subluxação dos componentes protéticos, ou quando abertura ligamentar patológica superior a 5 mm durante exame físico, ou frouxidão ligamentar superior a 5 mm nos testes de estresse em varo ou valgo durante exame físico ou nas radiografias anteroposteriores do joelho com carga), limitação de arco de movimento (quando menor que 50 graus associado a incapacidade física declarada pelo paciente), fratura periprotética, mal alinhamento, soltura asséptica, dor devido a não substituição da cartilagem patelar, desgaste do polietileno, fratura dos implantes, insuficiência do mecanismo extensor.

## Resultados

A infecção articular periprotética (IAP) foi a principal causa de RATJ, correspondendo a 47,73% dos casos. A soltura asséptica de um ou mais componentes representou a 2ª causa mais frequente de falha, acarretando 35,23% das revisões. Limitação do arco de movimento representou a 3ª mais frequente causa, sendo responsável por 5,68% das cirurgias. Instabilidade foi a 4ª causa mais frequente de falha, ocorrendo em 4,55% dos pacientes. As demais causas de revisão foram: fratura periprotética (3,41%), insuficiência por ruptura do mecanismo extensor (2,27%), dor atribuída a não substituição da cartilagem patelar (1,14%) ( Fig. 1 ).

**Fig. 1:** Distribuição das causas de falha da artroplastia total do joelho.

## Discussão

O principal achado do nosso estudo foi identificar que a principal causa de falha, em nossa série de artroplastias totais primárias do joelho, foi a infecção periprotética. Outras razões que conduziram à necessidade de revisão foram, em ordem decrescente, soltura asséptica, limitação do arco de movimento, e instabilidade articular. De modo similar, diversos recentes estudos demonstraram a infecção periprotética como o mecanismo de falha mais frequente das próteses primárias, alcançando de 20 a 37,7% das cirurgias. 2 23 24 25 No estudo de Evangelopoulos et al., 24 a infecção periprotética foi o principal mecanismo de falha com incidência de 26,3%. Entretanto, ao analisar apenas os pacientes submetidos à 2ª cirurgia de revisão, os autores identificaram que a incidência de infecção periprotética permanecia como motivo mais frequente de falha da ATJ com aproximadamente, 50% dos casos. Acreditamos que essa alta incidência de revisões por falha séptica observada em nosso trabalho possa ter sido influenciada, pelo menos parcialmente, pelo fato de as cirurgias terem sido realizadas em período de pandemia de covid-19. Nesse cenário, em diversos meses foram realizadas apenas cirurgias de urgência, como as infecções periprotéticas agudas e, consequentemente, posterior aumento da demanda reimplantação após tratamento de infecção em dois tempos.

A soltura asséptica representou o 2° motivo mais frequente de falha dos implantes em nossa série, ocorrendo em, aproximadamente, 35% dos casos. De modo similar, Bozic et al. 7 demonstraram que a infecção periprotética e soltura asséptica foram as duas razões mais frequentes para RATJ. Ao analisarem mais de 60 mil cirurgias primárias, os autores identificaram falha séptica em 25,1% dos pacientes e soltura asséptica em 16,7% dos casos. 7 Nossos resultados encontram-se em concordância com os de Koh et al. 26 e de Evangelopoulos et al., 24 que demonstraram a soltura asséptica como 2ª causa de revisão, acometendo, respectivamente, 32,7% e 25% das falhas. Siqueira et al., 1 ao analisar o registro de artroplastia de cinco países, identificaram a soltura asséptica como a primeira causa de revisão, acometendo 30% das cirurgias. Diversos outros estudos também relataram a soltura asséptica como principal mecanismo de falha de ATJ, com incidência variando entre 24 e 44%. 10 27 28 Identificamos que a incidência de soltura asséptica em nossa série apresenta-se em conformidade com a literatura, apesar de não ser o principal modo de falha dos nossos implantes.

A limitação do arco de movimento representou, em nossa série, a 3ª mais comum indicação de revisão de ATJ, com, aproximadamente 6% das cirurgias. De modo similar, no estudo de Le et al., a artrofibrose foi a terceira indicação mais frequente de revisão, muito embora a incidência apresentada corresponda a, aproximadamente, três vezes a observada em nosso estudo. Pietrzak et al. 11 estudaram apenas as causas assépticas de revisão e identificaram a rigidez articular como a segunda principal causa de revisão com incidência de 27,5%. Entretanto Le 29 e Koh et al. 26 demonstraram incidência que apenas 2,5% dos pacientes necessitaram de RATJ devido à limitação funcional imposta por restrição do arco de movimento. Tais diferenças podem ser explicadas, pelo menos parcialmente, pelas diversas definições de rigidez articular, assim como, ser influenciada pela demanda funcional e expectativas dos pacientes. 30 31 32 Devemos ainda observar que, em nossa série, incluímos apenas pacientes submetidos à revisão de componentes protéticos, desse modo, pacientes submetidos à manipulação sob narcose após ATJ ou mesmo à reoperação com liberação articular não foram analisadas, o que poderia ter modificado a incidência caso analisássemos reoperações após prótese do joelho. Em um estudo realizado no Brasil avaliando as complicações de curto prazo (até 1 ano), os autores identificaram que a rigidez articular foi a mais frequente complicação acometendo 7,5% dos pacientes submetidos à ATJ. 33

Em nosso estudo, a instabilidade dos componentes representou a 4ª causa de revisão de ATJ, com aproximadamente 5% das cirurgias. Outros estudos demonstraram incidência de instabilidade variando de 18,7 a 30%, configurando a 2ª ou 3ª mais frequente razão para revisão de ATJ. 10 28 29 Entretanto, nos trabalhos de Kasahara et al. 27 e Koh et al., 26 a instabilidade foi identificada em 9,3% e 6,5%, respectivamente. A instabilidade pós ATJ é, normalmente, indicada como causa de falha precoce, isto é, em implantes com menos de 2 anos de evolução. 6 11 Assim, a redução do número de cirurgias primárias eletivas realizadas no período do estudo, devido às restrições impostas pela pandemia de covid-19, pode justificar, pelo menos parcialmente, o número limitado de revisões devido à instabilidade pós ATJ. Outra explicação potencial pode ser atribuída ao tempo de espera pela realização desse tipo de cirurgia no serviço público de saúde brasileiro. Assim, eventuais casos iniciais de instabilidade podem potencialmente evoluir com soltura dos implantes, dificultando, assim, o diagnóstico primário de instabilidade dos componentes.

Apesar da importância de nossa série relatar a distribuição das diferentes causas de revisão de ATJ no cenário nacional, nosso estudo apresenta significativas limitações. Acreditamos que as restrições para realização de cirurgias eletivas impostas durante parte do período de pandemia de covid-19 podem ter tido influência sobre a incidência de alguns modos de falha, como por exemplo suspensão da realização de revisões assépticas eletivas em determinados períodos. Outra importante limitação refere-se à natureza retrospectiva de nossa análise. Assim, diversos pacientes submetidos à cirurgia de revisão haviam sido operados primariamente em outros hospitais. Deste modo, não tivemos controle sobre o tempo de evolução desde a implantação dos componentes até a ocorrência de falha com necessidade de cirurgia de revisão, tampouco, realizamos análises dos diferentes sistemas de próteses primárias implantadas. Schroer et al. 10 assinalam que 35,3% das artroplastias do joelho falham nos primeiros 2 anos após implantação, sendo mais frequentemente relacionados à fatores influenciados pelo cirurgião que pelo desempenho dos implantes. Entretanto, entendemos que tais informações são de alta relevância para o planejamento do sistema de saúde brasileiro diante do aumento do número de casos de RATJ, haja vista, os impactos quanto à necessidade de leitos hospitalares, recursos financeiros e treinamento técnico das equipes. De modo similar, acreditamos que o elevado número de cirurgias de revisão realizadas por falha séptica observado em nossa série, nos alerta para a necessidade de ampliação das medidas de prevenção dessas graves complicações.

Assim sendo, acreditamos que a adoção de medidas preventivas de infecção pós-operatória, tais como: otimização das condições clínicas pré-operatória, adoção de escores de estratificação de risco e investigação de pacientes com colonização por *Sthapylococcus aureus* resistente a meticilina possa impactar na redução do número de cirurgias de RATJ realizadas em nosso hospital.

## Conclusões

Identificamos a infecção articular periprotética como a mais frequente causa de RATJ. Os demais mecanismos de falha de ATJ foram, em ordem decrescente: soltura asséptica, limitação do arco de movimento e instabilidade. Fratura periprotética e insuficiência do mecanismo extensor foram causas menos frequentes de revisão de prótese primária em nossa série.
